# De novo transcriptome assembly from the nodal root growth zone of hydrated and water-deficit stressed maize inbred line FR697

**DOI:** 10.1038/s41598-023-29115-9

**Published:** 2023-02-03

**Authors:** Sidharth Sen, Shannon K. King, Tyler McCubbin, Laura A. Greeley, Rachel A. Mertz, Cheyenne Becker, Nicole Niehues, Shuai Zeng, Jonathan T. Stemmle, Scott C. Peck, Melvin J. Oliver, Felix B. Fritschi, David M. Braun, Robert E. Sharp, Trupti Joshi

**Affiliations:** 1 Institute for Data Science and Informatics, University of Missouri, Columbia, USA; 2 Department of Biochemistry, University of Missouri, Columbia, USA; 3 Division of Biological Sciences, University of Missouri, Columbia, USA; 4 Division of Plant Science and Technology, University of Missouri, Columbia, USA; 5 Department of Electrical Engineering and Computer Science, University of Missouri, Columbia, USA; 6 School of Journalism, University of Missouri, Columbia, USA; 7 Department of Health Management and Informatics, University of Missouri, Columbia, USA; 8 Interdisciplinary Plant Group, University of Missouri, Columbia, USA

**Keywords:** Sequence annotation, Genome assembly algorithms, Drought, Plant molecular biology

## Abstract

Certain cultivars of maize show increased tolerance to water deficit conditions by maintenance of root growth. To better understand the molecular mechanisms related to this adaptation, nodal root growth zone samples were collected from the reference inbred line B73 and inbred line FR697, which exhibits a relatively greater ability to maintain root elongation under water deficits. Plants were grown under various water stress levels in both field and controlled environment settings. FR697-specific RNA-Seq datasets were generated and used for a de novo transcriptome assembly to characterize any genotype-specific genetic features. The assembly was aided by an Iso-Seq library of transcripts generated from various FR697 plant tissue samples. The Necklace pipeline was used to combine a Trinity de novo assembly along with a reference guided assembly and the Viridiplantae proteome to generate an annotated consensus “SuperTranscriptome” assembly of 47,915 transcripts with a N50 of 3152 bp in length. The results were compared by Blastn to maize reference genes, a Benchmarking Universal Single-Copy Orthologs (BUSCO) genome completeness report and compared with three maize reference genomes. The resultant ‘SuperTranscriptome’ was demonstrated to be of high-quality and will serve as an important reference for analysis of the maize nodal root transcriptomic response to environmental perturbations.

## Introduction

Maize (*Zea mays* L.) is one of the most important food crops in the world. The United States (U.S.) is the largest producer of maize, with over 90 million acres dedicated to maize production^1^. In 2012, almost 78% of maize growing areas in the U.S. experienced drought conditions^2^, and in subsequent years, large regions have continued to face substantial drought events, resulting in sizeable yield losses. The socio-economic costs of drought are well recognized, and various organizations closely monitor its effects on food shortages both country- and world-wide^3^.

As such, there is major interest in understanding the effects of drought on the maize plant, in particular the growth and functioning of the root system because of its critical role in water uptake from the soil. Early studies reported that the nodal roots^4^, which develop from the base of the stem and produce the framework of the mature root system, can continue to grow under water stress conditions that inhibit the growth of the leaves and stem^5,6^. However very little is known about the physiology or genetics of the underlying mechanisms that give maize nodal roots this ability.

In plants, organ growth encompasses two types of cellular activity – cell division and cell expansion^7^. In primary and nodal roots of maize, cell division occurs in the apical 3 mm, whereas cell elongation occurs throughout the apical 10 mm^8^. The dynamics of both of these activities are altered in roots exposed to water stress^9^. The maize inbred line FR697, developed by Illinois Foundation Seeds Inc., was selected for studies of root growth responses to water stress because it exhibits a greater ability for primary, nodal and lateral root growth maintenance when compared to the reference inbred line B73 under similar water stress conditions^10–12^.

To gain insights into the genetic mechanisms behind drought adaptation or acclimation in maize nodal roots, *we utilized a diverse set of RNASeq libraries to generate a sufficiently diverse and large quantity of transcript reads to assemble a FR697 specific transcriptome to enable a detailed analysis of the effects of water deficit stress on the growth zones of nodal roots of the maize FR697 genotype.* Apical 1-cm samples of the root tips were collected from both B73 and FR697 plants grown under well-watered and water-deficit conditions in the field, and from FR697 in controlled-environment growth chambers^13^. The FR697 RNA-Seq paired-end samples generated from these experiments were combined with Iso-Seq transcript sequences obtained from an independent set of FR697 maize tissue samples to generate a de novo transcriptome assembly. This assembly was clustered with transcripts from a B73 reference genome guided assembly using the same set of RNA-Seq samples and the Viridiplantae proteome, to generate a consensus annotated set of “SuperTranscripts”, which are collectively called a “SuperTranscriptome”^14^. This dataset can be used as a surrogate for a FR697 reference genome, enabling various comparative studies with the reference genotype B73 and the nested association mapping (NAM) founder lines designed to explore the underpinning genetic networks that control nodal root growth responses under drought conditions.

## Materials and methods

### Iso-Seq transcriptome generation

Seeds of maize inbred line FR697 (http://www.maizegdb.org/cgi-bin/displaystockrecord.cgi?id=70667) were produced at the University of Missouri by self-pollination of plants from stocks originally obtained from Illinois Foundation Seeds Inc. (Tolono, IL, USA). A maize FR697 tissue collection was created using samples taken from various sections of plants grown under greenhouse conditions from these seeds. These samples comprised: unpollinated silks, immature tassel, immature ear, kernels 14 “days after pollination” (DAP), kernels 21 DAP, whole germinated kernels, whole seedling at the 2-leaf stage, young leaf, ligule, mature leaf-base, mature leaf-mid section, mature leaf-tip, sheath, nodal root minus tip, and nodal root tip. RNA was extracted from these tissue samples using the RNeasy (Qiagen, Hilden, Germany) kit with RLC buffer following the manufacturer’s recommended protocol. The RNA samples were then pooled for subsequent amplification, from which Barcoded SMRT libraries were prepared and sequenced on the PacBio platform with X SMRT cells by Novogene Corporation Inc. (Sacramento CA).

Resultant PacBio Iso-Seq reads were processed using the IsoSeq3 analysis pipeline (Pacific Biosciences)^15^. This included Circular Consensus Sequence (CCS) generation, full-length reads identification (“classify” step), clustering isoforms (“cluster” step), and a “polishing” step using the Arrow consensus algorithm. The resultant high-quality full-length PacBio isoforms were used as input for further steps.

### RNA-Seq sample collection and sequencing

**Figure 1:**
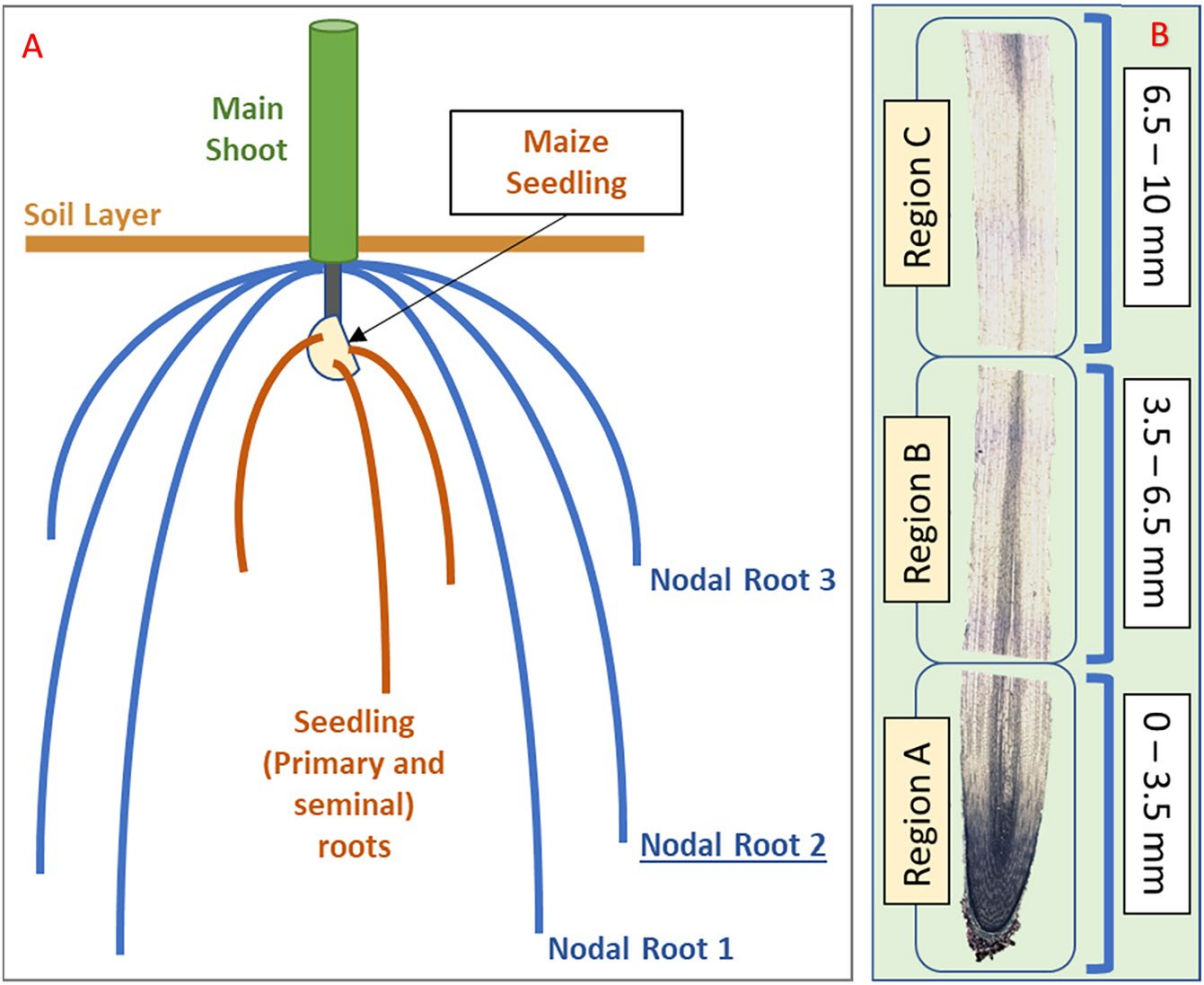
(**A**) Schematic diagram showing root growth in first 16 days after maize seedling germination, including the emergence of the primary and seminal roots, along with the first 3 Nodal roots. The first 10 mm tip of the nodal root 2 was collected and divided into 3 sections for RNA-seq library generation. (**B**) Maize nodal root no.2’s tip structure, divided into three sections: Region A, 0–3.5 mm; Region B, 3.5–6.5 mm; Region C, 6.5–10.5 mm.

Maize nodal root tip samples (node 2) (Fig. 1A) for RNA-Seq were collected from plants grown under two conditions – in the field (B73 and FR697) or in controlled-environment growth chambers (FR697). Samples collected from both growing conditions were sectioned into three regions (Fig. 1B) to address growth specific regional responses to water deficits^8^ at the following distances from the root apex: Region A, 0–3.5 mm (including the root cap); Region B, 3.5–6.5 mm; Region C, 6.5–10 mm.

Field experiments were performed at the Bradford Research Center, University of Missouri, Columbia, MO in 2017. B73 and FR697 seeds were planted at 12 seeds/m in 4.57 m long, four rows wide plots with 0.76 m row spacing in a randomized complete block design with six replications. Plants were grown to Vegetative-stage 3, which was 16 days after planting, equating to 33.2 growing degree days (GDD)^16^. The experiment was conducted under a rainout shelter, which allowed control over water availability by excluding precipitation. Well-watered plots were irrigated at regular intervals while water-stressed plots received no water after germination.

Growth chamber experiments were conducted using a split root system^10,13^ that was developed by the Sharp lab at the University of Missouri. This system consists of two concentric tubes that are used as inner and outer chambers to separate the seedling (primary and seminal) root system from the nodal root system, respectively, together with the substrate (PRO-MIX HP; Premier Tech, Québec, Canada) the roots are growing in. The substrate water potentials in each chamber were independently controlled by addition of pre-calibrated amounts of water. This system was used to sample nodal root tips from FR697 plants, with the intention of analyzing molecular and biochemical responses to two plant water stress levels: severe stress (− 0.9 MPa outer chamber, − 0.4 MPa inner chamber) and moderate stress (− 0.9 MPa outer chamber, well-watered inner chamber [≤ − 0.1 MPa]), together with a control treatment in which the substrate in both chambers was well-watered. Samples were collected 19 days after germination.

The nodal root tip sections were pooled into six biological replicates for field samples and five biological replicates for growth chamber samples. Each replicate contained a minimum of eight root sections representing a minimum of four plants. Root tips were taken from field and lab samples if their nodal root (node 2) lengths were within one standard deviation of the mean length within the batch from each treatment and genotype. Root tips were immediately frozen in liquid nitrogen and ground using a Qiagen/Retsch tissuelyser II bead-beater with 1/8″ stainless steel beads from Union Process (part#0070–01; Akron, OH). Root tip homogenate was then isolated using the RNeasy Plant Mini Kit (Qiagen). Isolated RNA was DNAse-treated with TURBO™ DNase (ThermoFisher Scientific, Waltham, MA), and quality was assessed using a 2100 Bioanalyzer (Agilent Technologies Inc.). Both these steps were carried out in the Sharp, Peck and Fritschi labs at the University of Missouri. Collected RNA samples were then sent to Novogene (Sacramento, CA) for library preparation and sequencing, producing high quality paired-end 150 bp RNA-Seq libraries. In total we used 79 RNA-Seq datasets, 34 from field-grown samples and 45 from growth chamber-grown samples. All methods, including cultivation and collection of plant material, were performed in accordance with relevant institutional, national, and international guidelines and legislation.

### SuperTranscriptome de novo assembly

**Figure 2:**
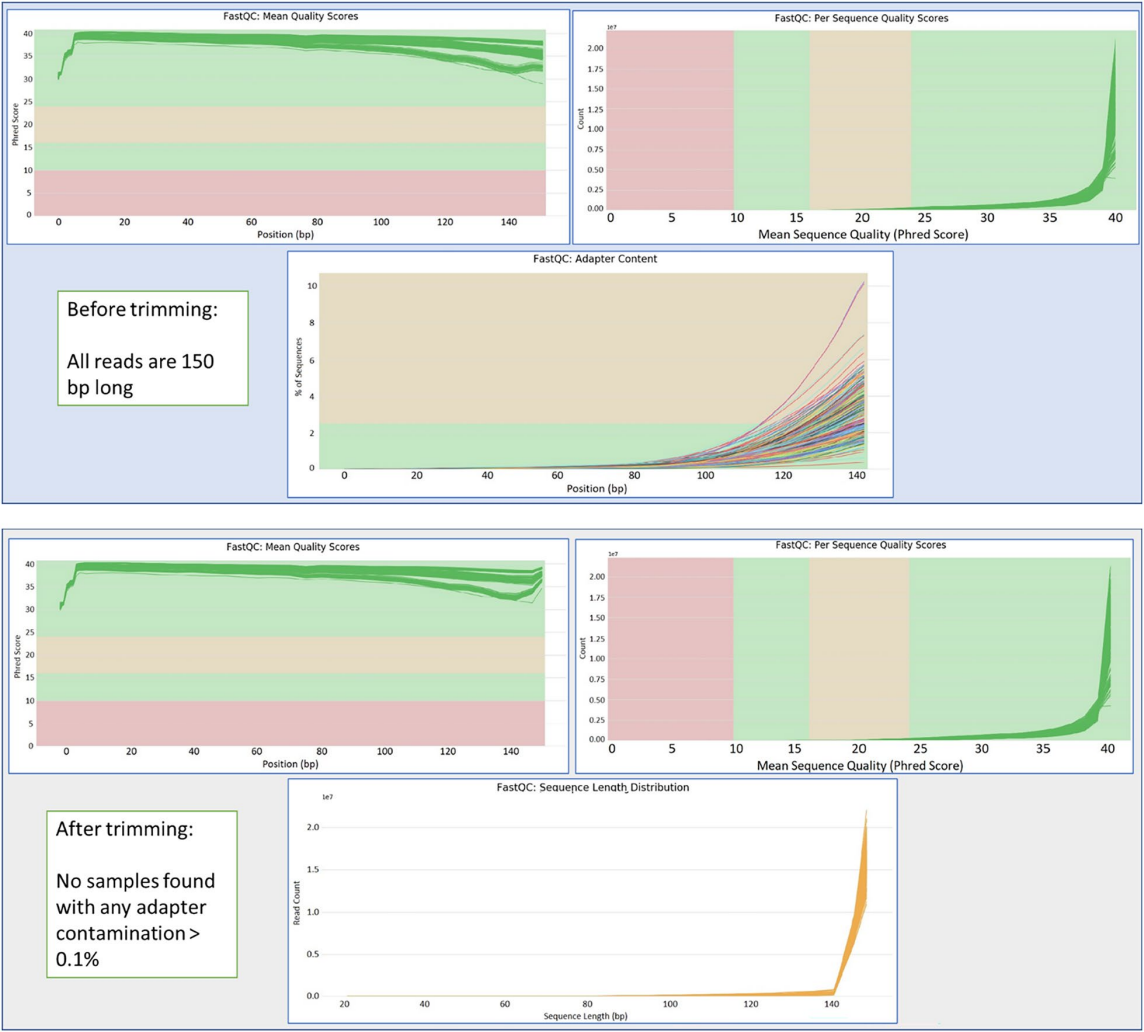
Quality assessment metrics before and after trimming the RNA-Seq samples. All raw reads were 150 bp long and after trimming, some adaptor sequence fragments were removed from the tail ends.

**Figure 3:**
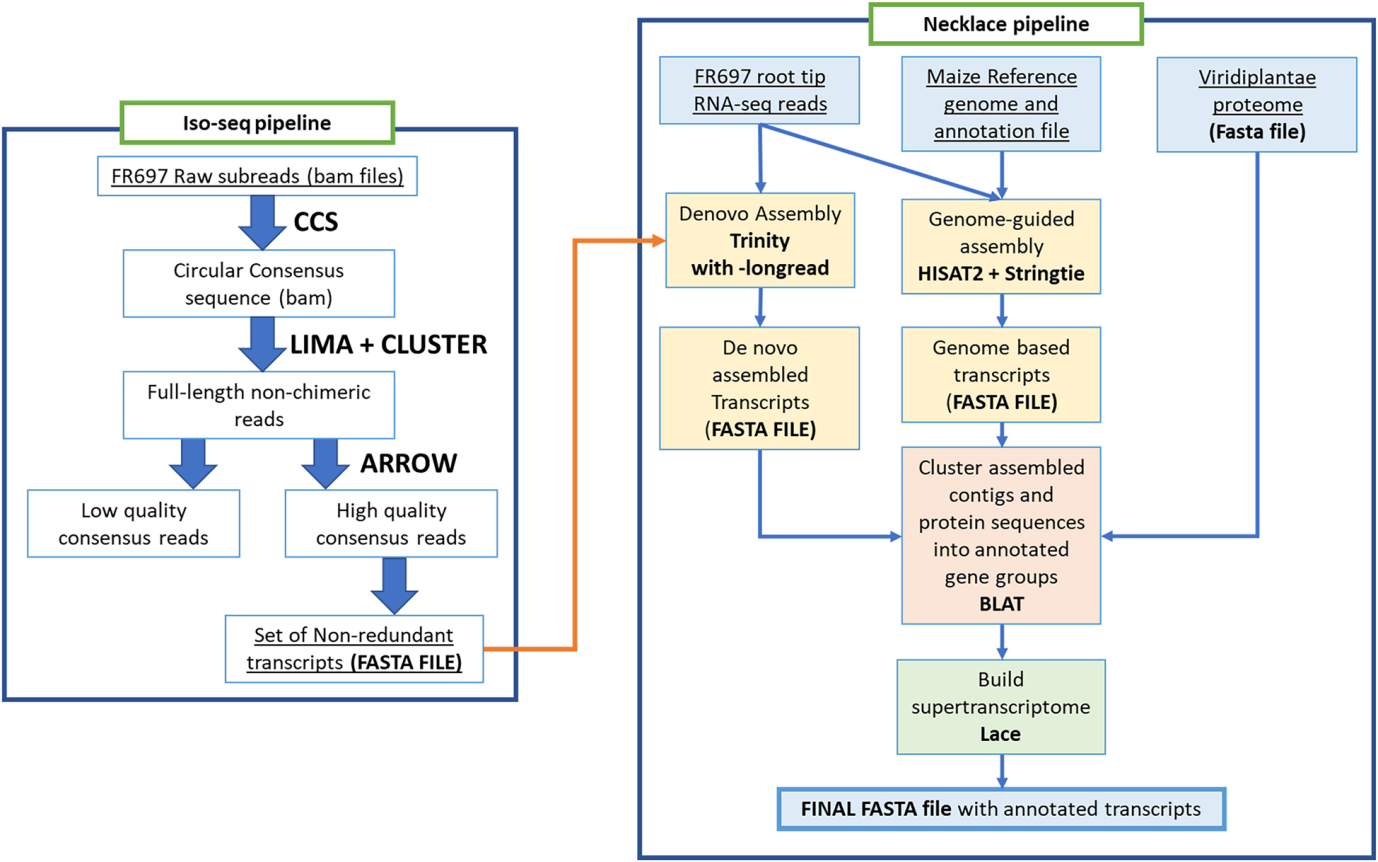
Overview of the steps to assemble the FR697 transcriptome. The first part consists of the Iso-Seq pipeline to generate a set of long read transcripts from Iso-Seq reads. These transcripts were used along with the RNA-Seq short read samples for a trinity transcriptome assembly. The trinity assembly along with a reference guided transcriptome assembly and the Viridiplantae proteome is combined by the Necklace pipeline, which generates a consensus “SuperTranscriptome” assembly, with the final output being a transcriptome fasta file with annotations.

The raw RNA-Seq reads were pre-processed by removing adapters and low-quality sequences using Trim-Galore (V. 0.6.4)^17^ with default settings and adapter auto detection. RNA-Seq read quality before and after trimming was assessed using FastQC^18^ and aggregated using MultiQC^19^, as presented in Fig. 2. FR697 SuperTranscripts were generated using the Necklace pipeline^20^. The pipeline consists of three major steps, as follows: (1) a de-novo transcriptome assembly with Trinity (v. 2.7.0)^21^ with “*longreads”* option to include the Iso-Seq transcripts; (2) a reference genome guided transcriptome assembly using the B73 v4 reference maize genome; and (3) the Viridiplantae clade proteome dataset to annotate the transcripts not included in the provided reference gtf/gff3 files. The fully modified pipeline used is presented in Fig. 3. We used the CD-HIT-EST program (v. 4.8.1)^22^ for three iterations with default parameters (similarity 95%) to reduce transcript redundancy in the Trinity assembly and to compare with the results of the Necklace pipeline.

### Gene ontology and assembly completeness analysis

To further annotate the SuperTranscripts identified by the pipeline, we used Blast2GO software^23^ to associate GO annotations to them. Blast2GO aligns sequences of interest to a given database of sequences. For our analysis the NCBI nr/nt nucleotide database was used. We selected the top blast hits for each SuperTranscript (blast E-value very close to 0), and reported the corresponding distributions of annotations for biological processes, molecular function, and cellular components.

To test the SuperTranscriptome assembly for completeness, we used the GenomeQC^24^ web application’s Benchmarking Universal Single-Copy Orthologs (BUSCO)^25^ implementation to search for conserved orthologous genes. GenomeQC was set to use the BUSCO dataset embryophyte_odb9 (plants) along with AUGUSTUS^26^ species “maize”, and the option to compare the results against the precomputed results of three maize reference genomes –

1. “MaizeB73_v4_scaffolds”(https://www.maizegdb.org/genome/assembly/Zm-B73-REFERENCE-GRAMENE-4.0) ,
2. “MaizeMo17_CAU_scaffolds” (https://www.maizegdb.org/genome/assembly/Zm-Mo17-REFERENCE-CAU-1.0), and
3. “MaizeW22_NRgenes_con” (https://www.maizegdb.org/genome/assembly/Zm-W22-REFERENCE-NRGENE-2.0).

## Results and technical validation

### SuperTranscript annotation and verification

**Figure 4:**
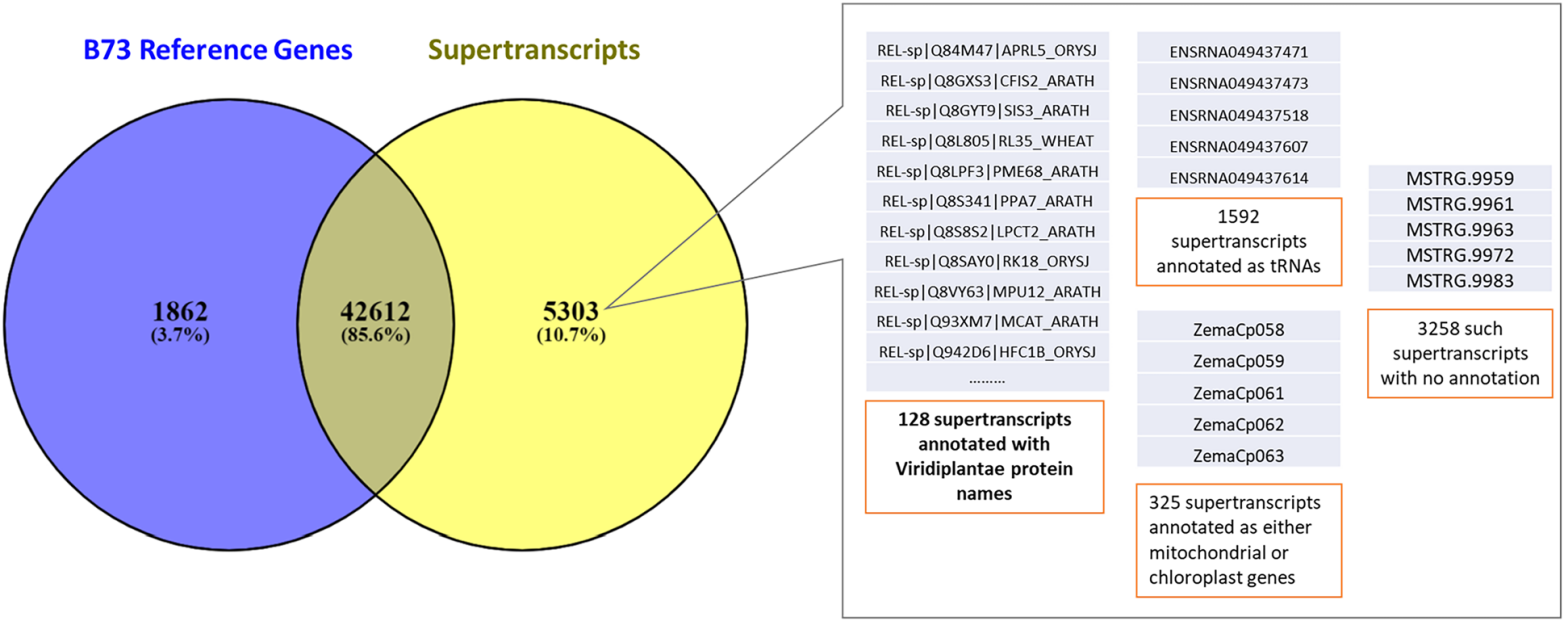
Venn Diagram showing that 42,612 SuperTranscripts identified and annotated with corresponding maize reference gene IDs. Of the remaining 5303, 128 SuperTranscripts were predicted to be coding for certain proteins from the Viridiplantae proteome, 1592 were annotated as tRNAs, 325 were annotated as either mitochondrial or chloroplast genes, and the remaining 3258 were unidentified.

The intermediate trinity de-novo transcriptome assembly was generated with an ExN50 maximum value of 2173 at Ex = 95 and transcripts = 38,512 (ExN50 is the maximum length of assembled contigs/scaffolds which captures Ex% of total gene expression^27^). The intermediate genome guided transcriptome assembly using HISAT2 reported an alignment rate of 84.08% for the RNA-Seq reads to the maize genome. Finally, the Necklace pipeline produced 47,915 unique SuperTranscripts. Of the total, 42,612 were assigned unique maize reference gene IDs by the pipeline. Of the remaining 5303 SuperTranscripts, 1592 were annotated as tRNAs, 325 as a combination of mitochondrial and chloroplast genes, 3258 as novel unknown transcripts, and 128 were predicted to transcribe for proteins found in the Viridiplantae proteome, suggesting FR697 genotype-specific novel genes (Fig. 4). The N50 of the assembled SuperTranscripts was significantly improved compared to trinity transcripts, from 1589 to 3152, which is close to the average size of maize genes of 4 Kb^28,29^. The number of redundant transcripts was also significantly reduced when compared to the original trinity assembly and the results of CD-HIT-EST after three iterations (Table 1).

**Table 1:** Various metrics for the FR697 SuperTranscriptome compared to three maize reference genotypes and the improvement of annotation over the initial trinity assembly.

	MaizeMo17_CAU_scaffolds	MaizeB73_v4_scaffolds	MaizeW22_NRgene_con	Trinity Transcripts	Trinity with cd-hit 3 times	FR697 SuperTranscripts
Number of scaffolds	2560	596	306	720,299	540,032	47,935
Total size of scaffolds	2,182,615,441	2,134,339,606	2,133,868,603	587,326,543	402,421,838	98,256,966
Total scaffold length as percentage of assumed genome size	99.20979277	97.01543664	96.99402741	24.47193929	16.76757658	4.09404025
useful amount of scaffold sequences (≥ 25 K nt)	2,166,421,525	2,134,248,774	2,132,523,330	58,570	58,570	0
% of estimated genome that is useful	98.47370568	97.01130791	96.93287864	0.002440417	0.002440417	0
Longest scaffold	32,176,138	39,317,442	83,688,764	29,921	29,921	22,234
Shortest scaffold	1007	5568	711	176	182	32
Number of scaffolds > 1 K nt	2560	596	305	149,963	97,893	31,091
Number of scaffolds > 10 K nt	2216	591	291	510	320	271
Number of scaffolds > 100 K nt	475	366	130	0	0	0
Number of scaffolds > 1 M nt	304	296	97	0	0	0
Number of scaffolds > 10 M nt	69	69	62	0	0	0
N50	10,204,498	10,679,169	35,520,101	1589	1336	3152
L50	69	62	19	95,173	72,937	9621
NG50	9,989,738	10,214,929	33,636,442	0	0	0
LG50	70	66	20	0	0	0
% A	26.16202361	26.17515251	26.11362927	24.76932496	24.77012567	24.91816509
% C	23.04310739	23.08858481	22.92158314	25.33622867	25.32836998	24.42904761
% G	23.03462569	23.10360491	22.93553555	25.01578785	24.9863945	24.9521983
% T	26.15118015	26.1942841	26.12596901	24.87865851	24.91510985	25.70053812
Total number of Ns	35,119,661	30,699,779	40,613,559	0	0	50
% N	1.609063161	1.438373674	1.90328303	0	0	5.09E-05

N50: sequence length of the shortest contig at 50% of the assembly size; L50: Number of contigs/scaffold whose length sum equals half of genome size; NG50: sequence length of the shortest contig at 50% of the full genome assembly length—not calculated for transcriptome assembly; LG50: Number of contigs/scaffold whose length sum equals half of whole genome assembly length -also not calculated for transcriptome assembly.

Blastn^30,31^ was used to compare the maize gene IDs annotated SuperTranscripts to the actual coding region sequences of maize genes in the B73 genome. All annotated SuperTranscripts were found to be in the top three blastn hits and within 93% identity threshold of maize genes with the same IDs. We then used HISAT2 to align a representative subset of the nodal root RNA-Seq samples against the assembled SuperTranscriptome. The alignment rate averaged around 85% for all samples. This was a significant increase from an average of 80% alignment rate when the same samples were aligned against the reference B73 v4 genome.

### GO annotation for SuperTranscripts coding for Viridiplantae proteins

**Figure 5:**
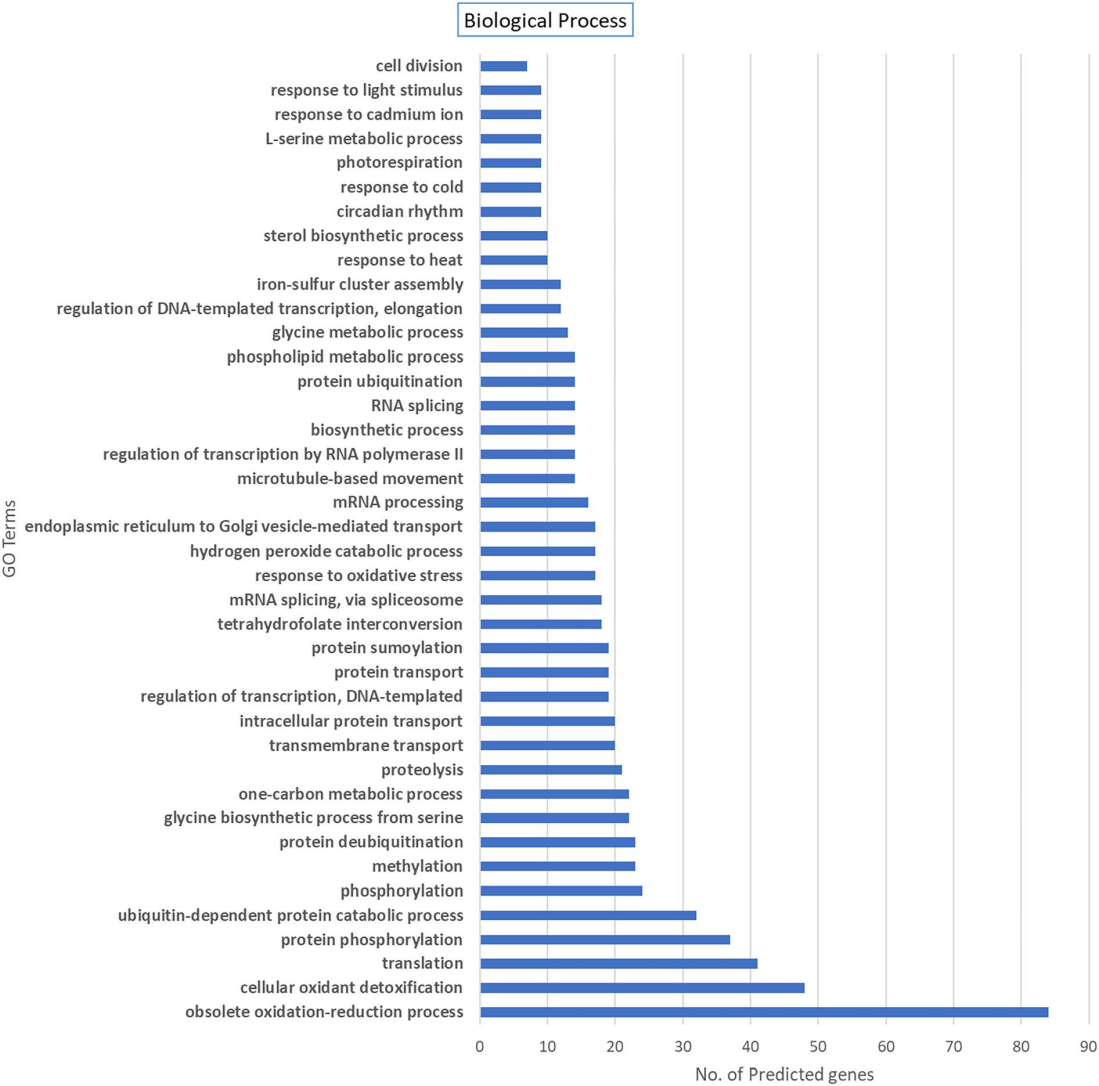
GO annotations distribution for the 128 SuperTranscripts assigned by Blast2GO^28^. This figure shows the associations of these sequences to the Biological Processes domain for GO terms. Majority of the SuperTranscripts are categorized under the “obsolete oxidation–reduction process” term.

**Figure 6:**
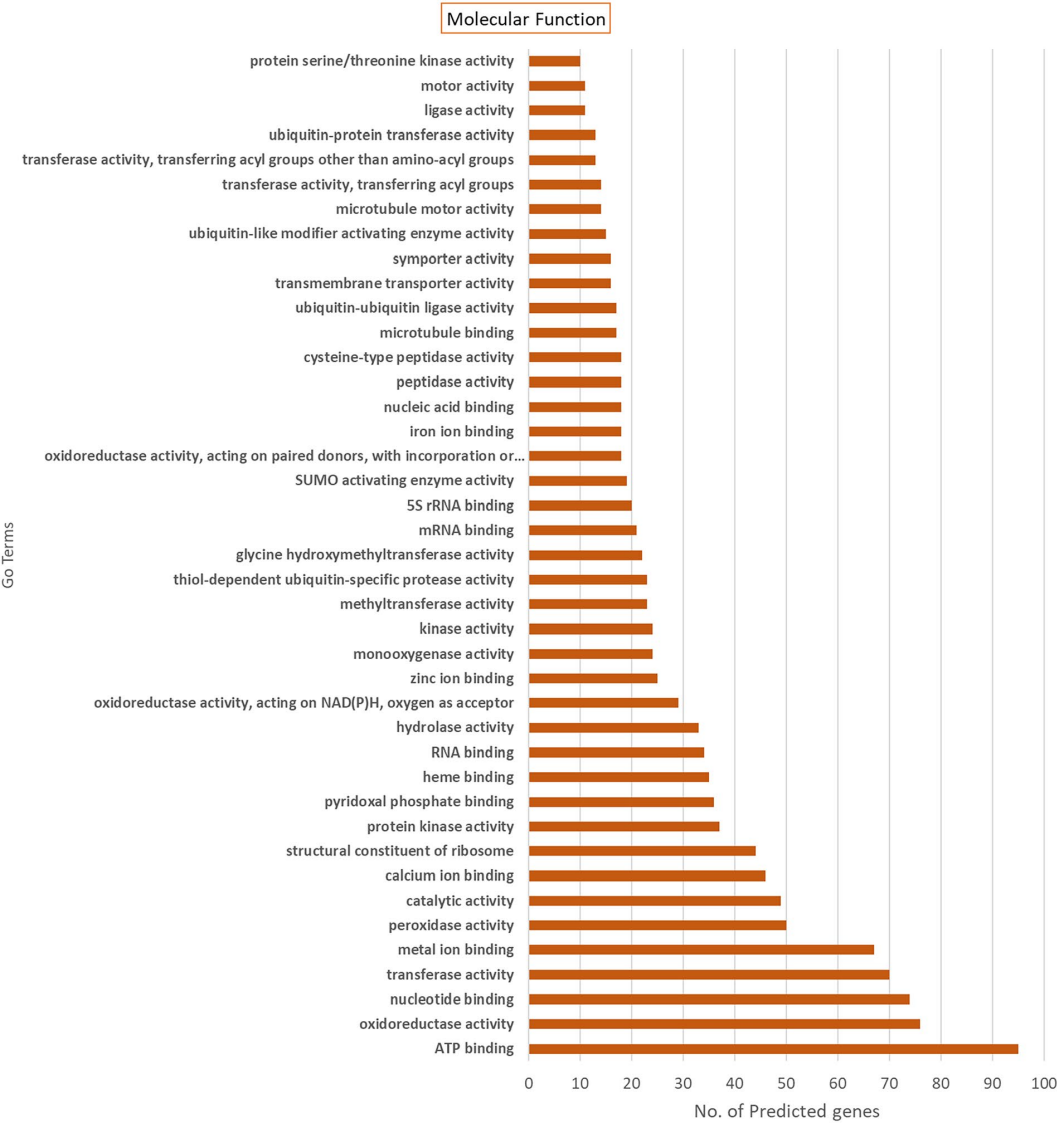
GO annotations distribution for the 128 SuperTranscripts assigned by Blast2GO^28^ showing the associations to the Molecular Function domain for GO terms. It seems that a majority of the SuperTranscripts are associated with ATP binding activity as shown by the assigned GO term.

**Figure 7:**
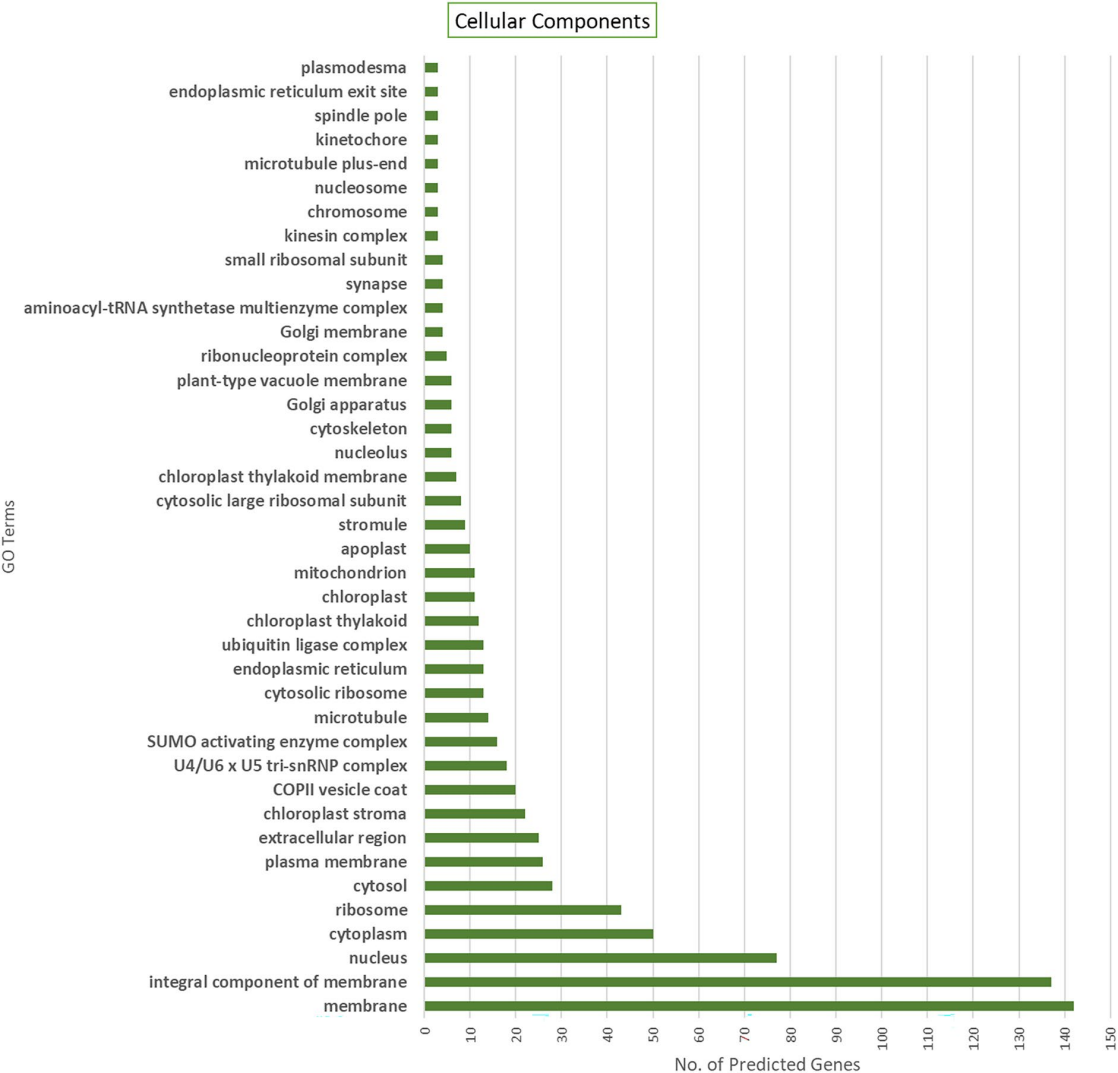
GO annotations distribution for the 128 SuperTranscripts assigned by Blast2GO^28^. This figure shows the associations of these sequences to the third domain of GO terms – Cellular Components. Majority of the SuperTranscripts seem to be associated with the terms – “integral component of membrane” and “membrane”.

The 128 SuperTranscripts identified to code for Viridiplantae proteins were annotated with GO terms using the Blast2GO software. For annotations within the GO Biological processes domain (Fig. 5), we report coverage across a range of terms associated with transport of molecules (terms such as protein transport, transmembrane transport). We also see a significant number of SuperTranscripts associated with terms for responses to various environmental changes, especially response to heat and cold along with oxidative stress which suggest that these SuperTranscripts are key players in root growth maintenance seen in the FR697 genotype. For the Molecular Function GO domain (Fig. 6) – we note that a significant number of SuperTranscripts were associated with various ion binding functions such as “iron ion binding”, “zinc ion binding”, etc. This suggests a role of these SuperTranscripts in pathways related to Ion transport which occurs via cell membrane and is an integral part of nutrient uptake in roots. For the Cellular Component GO domain (Fig. 7), we see a significant number of Supertranscripts connected with terms related to microtubules, which are known to have a role in cell division. The major terms in this domain are “Membrane” and “Integral component of Membrane”, which in taken together with the annotations of ion binding and transport from the Molecular Function GO Domain – again suggest that many of these SuperTranscripts play a role in nutrient uptake and homeostasis.

### Assembly completeness analysis

**Figure 8:**
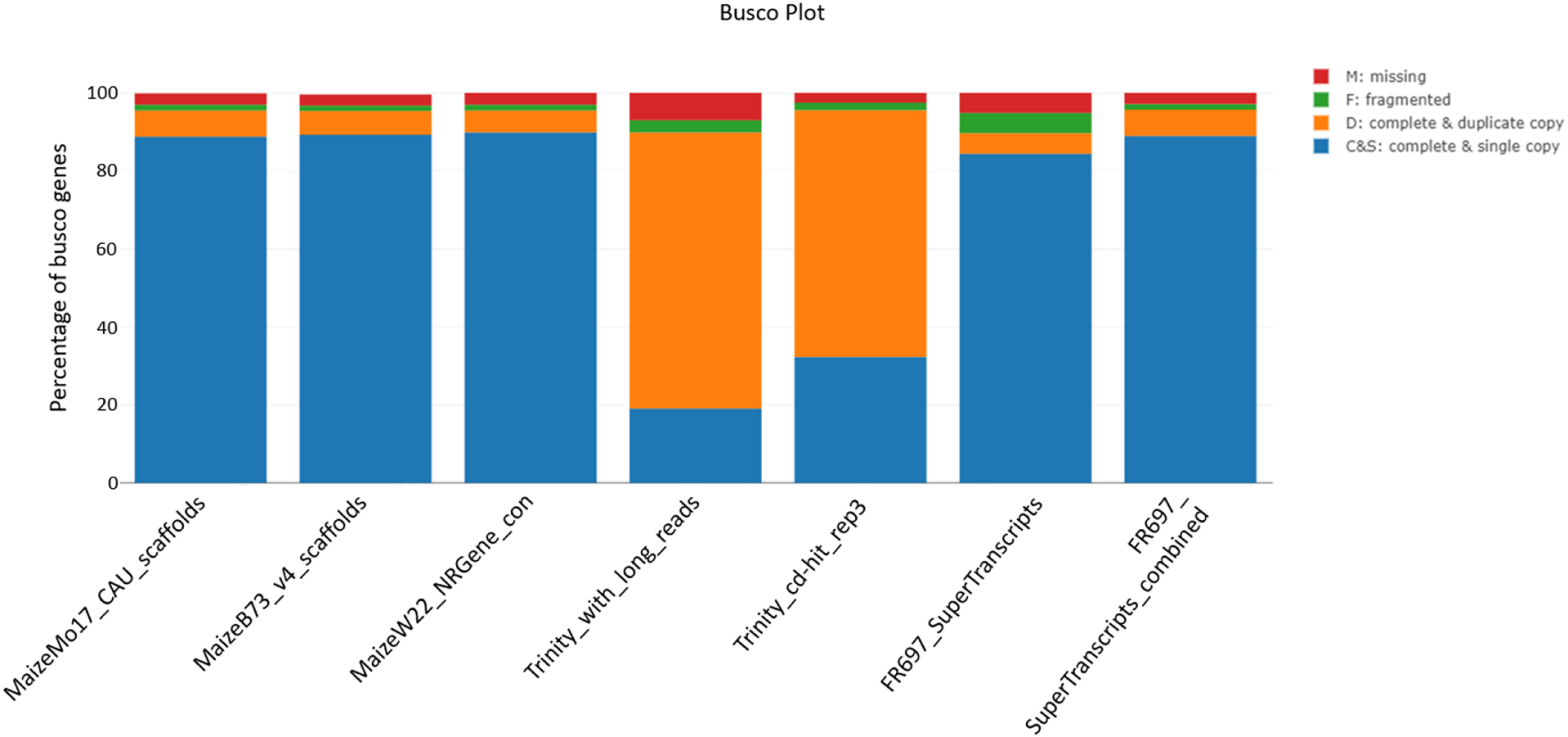
BUSCO analysis of “FR697 combined SuperTranscriptome” (full maize gene sequences replacing their annotated SuperTranscriptome counterparts) compared against the unmodified SuperTranscriptome, the first Trinity assembly using the Iso-Seq long reads, trinity assembly after 3 rounds of CD-HIT transcript redudancy reduction and 3 maize reference genome datasets.

As our goal was to see if any unique genes or transcripts are present in the FR697 genotype compared to B73, we replaced the maize id annotated SuperTranscripts with their respective full maize genes. For this, 42,612 full maize genes replaced their corresponding annotated SuperTranscripts from the assembly along with the 5303 assembled transcripts, resulting in the “FR697 combined SuperTranscriptome”. This dataset, along with the original SuperTranscriptome assembly, was analyzed by GenomeQC, and the generated BUSCO results were compared against the previous datasets, as presented in Fig. 8, reporting a similar completed score of about 95% (C&S, D sections of the barplot). We also compared the BUSCO results for the initial trinity transcript assembly, and the results of transcript redundancy reduction by CD-HIT-EST for an objective comparison of how many redundant and duplicate transcripts was reduced by the assembly pipeline.

## Conclusion

In this work we present an annotated de novo transcriptome assembly resource to characterize the inbred FR697 maize genotype known to show a superior ability for root growth maintenance under water-stress conditions. The assembly comprises a library of gene length SuperTranscripts, which were annotated as *Zea mays* protein-coding genes using the Necklace pipeline. This pipeline also used a reference genome guided transcriptome assembly along with the Viridiplantae proteome to assign gene IDs to specific SuperTranscripts. From a total of 47,915 unique SuperTranscripts, 42,612 were assigned unique maize gene IDs by the pipeline, 1592 were annotated as tRNAs, 325 as a combination of mitochondrial and chloroplast genes, and 128 were predicted to transcribe for proteins found in the Viridiplantae proteome, suggesting FR697 genotype-specific novel genes. As our final goal was to generate a reference gene set as close as possible to a complete assembly, we replaced the maize gene id annotated SuperTranscripts with their corresponding full maize gene sequences while retaining the remaining 5303 SuperTranscript sequences, thus generating what we term the “FR697 combined SuperTranscriptome”. Both datasets were then assessed for completeness by comparing to three different maize reference genomes and were found to be of similar quality. We also intend to follow up this study by collecting more batches of nodal root tips from plants grown at various water stress levels to study the gene expression landscape of root growth maintenance for FR697. This SuperTranscriptome will allow us to assemble and quantify transcripts from this expanded study and do an objective comparison against the gene expression profile for B73 samples grown in similar conditions. It is anticipated that this dataset will provide a valuable resource to understand the ability of maize roots to maintain elongation under water stress, and to gain insights into the mechanisms of such adaptation in other plant species.

## Data Availability

The datasets were submitted to NCBI under the bioproject ID: “PRJNA719429: De-novo transcriptome assembly from the nodal root growth zone of maize inbred line FR697”. The RNA-seq reads used in the assembly were submitted to the NCBI Sequence Read Archive database under the submission ID—SRP313297^32^, Iso-Seq reads under submission ID—SRX10509801^33^, and the FR697 transcriptome assembly to the NCBI Transcriptome Shotgun Assembly (TSA) database under submission ID—GJCA00000000.1^34^ (access under Genbank:Nucleotide). The IDs in the fasta headers were slightly modified to fit the TSA standard. The original de novo transcriptome assembly fasta file generated by the Necklace pipeline with the unmodified headers was deposited in figshare – “https://doi.org/10.6084/m9.figshare.14332364.v1”. Datasets which include the 128 protein coding transcripts, their GO annotation and the merged transcriptome data were also deposited in the same figshare repository as above.

## Code availability

Scripts used to process and generate some of the figures along with the main configuration file for the Necklace pipeline is available at this GitHub repository—https://github.com/sid5427/supertranscriptome_assembly . Also included are the log files for the Trinity assembler and CD-HIT analysis – both of which contain the commands and parameters as used. These software tools and their corresponding versions were used for this study. Most were run using default parameters and any changes are noted. IsoSeq v3.2 Pipeline—https://github.com/PacificBiosciences/IsoSeq. Fastqc v0.11.9—https://www.bioinformatics.babraham.ac.uk/projects/fastqc/. Trim-galore v0.6.2- https://www.bioinformatics.babraham.ac.uk/projects/trim_galore/. MultiQC v1.6—https://multiqc.info/. Necklace V1.11- https://github.com/Oshlack/necklace. Trinity v2.8.5—https://github.com/trinityrnaseq/trinityrnaseq. HISAT2 v2.2.0—http://daehwankimlab.github.io/hisat2/. StringTie v2.1.4—https://ccb.jhu.edu/software/stringtie/. BLAT v36—https://genome.ucsc.edu/goldenpath/help/blatSpec.html. Lace v1.14.1—https://github.com/Oshlack/Lace. GenomeQC—https://genomeqc.maizegdb.org/. Blast2GO v5.2.5—https://www.blast2go.com/.
